# First Morning Pregnanetriol and 17-Hydroxyprogesterone Correlated Significantly in 21-Hydroxylase Deficiency

**DOI:** 10.3389/fendo.2021.808254

**Published:** 2022-01-24

**Authors:** Tomoyo Itonaga, Masako Izawa, Takashi Hamajima, Yukihiro Hasegawa

**Affiliations:** ^1^ Division of Endocrinology and Metabolism, Tokyo Metropolitan Children’s Medical Center, Tokyo, Japan; ^2^ Department of Pediatrics, Oita University Faculty of Medicine, Oita, Japan; ^3^ Department of Pediatric Endocrinology and Metabolism, Aichi Children’s Health and Medical Center, Aichi, Japan

**Keywords:** Urinary pregnanetiol, 17-hydroxyprogesterone, 21-hydroxylase deficiency, congenital adrenal hyperplasia, first morning urine sample, therapy monitoring, glucocorticoid

## Abstract

**Background:**

Biochemically monitoring 21-hydroxylase deficiency (21-OHD) is challenging. Serum/blood 17-hydroxyprogesterone (17OHP) measurements are normally used for this purpose. Urinary pregnanetriol (PT), a urinary metabolite of 17OHP, may also be used. Based on auxological data, we previously reported that the optimal first morning PT value fell in the range of 2.2–3.3 mg/gCr (95% confidence interval of the mean) and 0.59-6.0 mg/gCr (10^th^ – 90^th^ percentile) for monitoring 21-OHD treatment. No report thus far has directly compared the first morning urinary PT value with the 17OHP value at various times during the day.

**Objective:**

To explore the correlation between the first morning urinary PT value before glucocorticoid administration and the serum/blood 17OHP value at three time points, namely, before and two and four hours after glucocorticoid administration.

**Design:**

This was a prospective study done at two children’s hospitals.

**Methods:**

In total, 25 patients with 21-OHD aged 3-25 years were recruited. Their urinary PT levels and 17OHP levels were measured for three days within a total period of one week. The first morning PT value was collected on all three days. Dried blood spots and serum were used to measure 17OHP.

**Results:**

The range for the first morning PT value for all the samples (n=69) was 0.10-56.1 mg/gCr. A significant, positive correlation was found between the first morning PT and 17OHP values before medication (r=0.87, p<0.01), and weaker correlation was observed between the first morning PT and 17OHP values after medication.

**Conclusions:**

The first morning PT correlated more significantly with 17OHP before the morning medication. Measuring the first morning PT value may be more practical and useful for monitoring 21-OHD biochemically.

## Introduction

The most common form of congenital adrenal hyperplasia, 21-hydroxylase deficiency (21-OHD), is an autosomal recessive disease caused by mutations in *CYP21A2* and has an incidence of 1:15,000-18,000 births (1, 2). Insufficient cortisol synthesis in patients with 21-OHD leads to an impaired negative feedback drive and increased ACTH secretion, resulting in excess 17OHP and adrenal androgens. Glucocorticoid (GC) therapy for children with 21-OHD aims to compensate for the cortisol deficiency and suppress excess adrenal androgen production (3, 4). In childhood, excess androgens lead to masculinization in females. Increased height velocity and acceleration of bone maturation are observed regardless of sex. The abnormal rate of bone maturation results in loss of growth potential and short stature.

Calibrating the medications for 21-OHD is difficult. The gold standard of monitoring of 21-OHD is auxological data, such as height, body weight, and bone age, which require a long time period (months to a year) to evaluate (5–7). Theoretically, biochemical monitoring using serum 17OHP, a substrate of 21-OH, and pregnanetriol (PT), its urinary metabolite, allows a much shorter monitoring period (hours to days). However, in the clinical setting, monitoring with 17OHP is challenging because the target range for disease control is not well-defined (3, 4, 8). The target for 17OHP is reportedly 4-12 ng/mL before early morning medication, but this information is not based on auxological data (9, 10). Our previous studies demonstrated that morning urine PT can be used as an index of control in prepubertal patients with 21-OHD based on height velocity, body weight, and bone age (11, 12). According to these studies, 2.2-3.3 mg/gCr (95% confidence interval (CI) for the mean) and 0.59-6.0 mg/gCr (10th - 90th percentile) were proposed as the optimal range for the first morning urinary PT value (12).

Measuring urinary steroid metabolites, including PT, is noninvasive and is more useful for periodic, repetitive measurements than measuring serum/blood 17OHP, which is invasive and varies highly depending on when the samples are taken (10). Furthermore, 17OHP measurements vary depending on the method used. Commonly used immunological assays are known to have cross-reactivity for other steroids, such as 17-hydroxypregnenolone; thus, liquid chromatography-tandem mass spectrometry (LC-MS/MS) is recommended internationally (3).

The present study aimed to explore the correlation between the first morning urinary PT value before glucocorticoid administration (0h-PT) and the serum and blood 17OHP values at three time points, namely, before (0h-17OHP) and two and four hours after medication (2h-17OHP, 4h-17OHP), with 17OHP used as a reference for the LC-MS/MS value.

## Patients and Methods

### Patients

In total, 25 patients with 21-OHD aged 3-33 years who were followed up at Tokyo Metropolitan Children’s Medical Center and Aichi Children’s Health and Medical Center were recruited after obtaining their informed consent (**Supplementary Table 1**). The inclusion criteria were as follows:

1. Well-defined classic and non-classic 21-OHD (3, 4); 21-OHD was diagnosed on the basis of elevated 17OHP and the patient’s urinary steroid metabolic profile as determined by gas chromatography-mass spectrometry (GC-MS) analysis.
2. Oral GCs, including hydrocortisone (HDC), dexamethasone (DEX), and prednisolone, administered as treatment for 21-OHD only.
3. No change in GC administration for one month before study commencement and during the study.
4. Availability of early morning, first urine sample

Of the 25 patients enrolled, 24 had the classic phenotype. The one, remaining patient had the non-classic phenotype. As replacement therapy, 17 and 8 patients received HDC in three divided doses and DEX once daily, respectively. In addition to GCs, fludrocortisone was administered to 23 of the 24 patients with the classical phenotype.

### Protocol

Morning urine samples for measuring PT and serum/blood samples for measuring 17OHP taken at the three times points described above were collected for three days within a period of one week (**Table 1**). On day 1, morning urine sampling (0h-PT) was done at home, and dried blood spots were collected at the hospital using filter paper (DBS) at 2 and 4 hours after GC administration (2h-17OHP, 4h-17OHP). On days 2-3, morning urine sampling (0h-PT) and DBS before GC administration (0h-17OHP) were done at home. Thus, the 0h-PT values were taken on all three days. DBS was collected for 2h-17OHP and 4h-17OHP on day 1 and for 0h-17OHP on days 2 and 3. In addition, serum sampling at two hours after GC administration (2h-17OHP) for measuring 17OHP was performed at the hospital to compare the LC-MS/MS and immunoassay results on day 1 (**Table 2**).

**Table 1:** Protocol.

Day	Day 1			Day 2	Day 3
	Before morning administration	2 hours after administration	4 hours after administration	Before morning administration	Before morning administration
**Time**	7:00-8:00 a.m.	9:00-10:00 a.m.	11:00-12:00 a.m.	7:00-8:00 a.m.	7:00-8:00 a.m.
**Sampling place**	Home	Hospital	Hospital	Home	Home
**Sample types**					
** Urine**	(1)	–	–	(2)	(3)
** Dried blood spot**	–	(4)	(5)	(6)	(7)
** Serum**	–	(8)	–	–	–

**Table 2:** PT and 17OHP values.

	Range (mean)
**First morning PT (0h-PT), mg/gCr**	
Day 1 (n=25)	0.12-56.1 (9.14)
Day 2 (n=24)	0.10-32.7 (6.95)
Day 3 (n=23)	0.12-41.3 (7.56)
Additional day* (n=2)	1.96-7.80
**17OHP before oral GC (DBS 0h-PT), ng/mL**	
Day 2 (n=24)	0.28-98.1 (28.4)
Day 3 (n=22)	0.63-99.0 (29.4)
Additional day* (n=2)	1.73-34.0
**17OHP 2 hours after oral GC (2h-17OHP), ng/mL**	
DBS 17OHP (n=23)	0.44-77.1 (12.9)
Serum 17OHP by ELISA (n=24)	0.30-126 (23.6)
Serum 17OHP by LC-MS/MS (n=23)	0.14-71.6 (13.1)
**17OHP 4 hours after oral GC (4h-17OHP), ng/mL**	
DBS 17OHP (n=23)	0.30-87.2 (12.8)
**Other data from venous blood at 2 hours after oral GC**	
Cortisol^†^, µg/dL (n=15)	4.3-43.5 (18.5)
ACTH, pg/mL (n=23)	<2.0-466 (106)
Plasma renin activity, ng/mL/hr (n=24)	0.2-11 (4.1)

*Two patients agreed to provide samples for one more day.

^†^Only patients receiving hydrocortisone (HDC).

The patients or their guardians were instructed in how to perform DBS sampling on day 1. The instructions were as follows: 1) Disinfect fingertip. 2) Prick the disinfected finger with a clean puncture needle. 3) Use filter paper to absorb the blood, completely filling the two, 1 cm-diameter circles. 4) Dry the filter paper at room temperature, place it in a plastic bag, and send it to the hospital.

Samples were not taken for two weeks following any experience of stress requiring additional GC administration.

### Sample Analysis

Urinary PT (0h-PT) was measured by GC-MS using an QP2010 mass selective detector (Shimadzu, Kyoto, Japan) with an Ultra ALLOY-5 stainless steel capillary column (Frontier Laboratories, Fukushima, Japan). The sensitivity was 0.01 mg/L. Blood 17OHP in the DBS (0h-, 2h- and 4h-17OHP) was measured by enzyme linked immunosorbent assay (ELISA) (Siemens Healthcare Diagnostics, Tokyo, Japan) after steroid extraction. The 17OHP values were measured twice in both spots, and the average of the two values was calculated. Serum 17OHP (2h-17OHP) was measured using ELISA (IBL International GmbH, Hamburg, Germany) and LC-MS/MS (ASKA Pharmaceutical Medical Corporation, Kanagawa, Japan). Approximately 20 μL of each sample after extraction and derivatization, as described previously (13), was analyzed for LC-MS/MS using a Nexera LC system (Shimadzu, Kyoto, Japan) equipped with an API 4000 triple quadrupole mass spectrometer (Sciex, Framingham, MA, USA) on positive ion mode. The separation of the steroids in the samples was carried out using a Kinetex C18 column (1.7 µm, 2.1 × 150 mm i.d.; Phenomenex, CA, USA) with a flow rate of 0.5 mL/min at 50°C. The selected reaction monitoring (SRM) method was used to detect 17OHP. The lower limit of quantification was 50 pg/mL.

The 2h-17OHP value on day 1 was measured using all the methods, namely DBS 17OHP with ELISA and serum 17OHP with ELISA and LC-MS/MS. Next, the correlation between the DBS 17OHP and serum 17OHP values (using by ELISA and LC-MS/MS) was analyzed. Based on the results, the DBS 17OHP values were converted into serum 17OHP values (by ELISA and LC-MS/MS).

### Statistical Analysis

Pearson or Spearman correlation and regression analysis was used to assess for a relationship between each of the following pairs: 1) 0h-PT and DBS 17OHP; 2) 0h-PT and DBS 2h-17OHP; 3) 0h-PT and DBS 4h-17OHP; 4) DBS 17OHP (ELISA) and serum 17OHP (ELISA); and 5) DBS 17OHP (ELISA) and serum 17OHP (LC-MS/MS). The value for each pair was measured on the same day. P <0.05 indicated statistical significance. All statistical analyses were performed using Easy R version 3.4.1 (14). Finally, the optimal range was calculated based on past reports (9, 10, 12) using the regression formula described above.

### Ethics Statement

The present study was conducted in accordance with the 1964 Helsinki Declaration and its later amendments (in 2013) or comparable ethical standards. This study was approved by the Institutional Ethics Committee of Tokyo Metropolitan Children’s Medical Center (No. H29b-176). Written informed consent for participation was obtained from the patients and/or their legal guardians.

## Results

### Regression Analysis of the Measurement Methods

There was a significant correlation between the DBS 17OHP by ELISA and serum 17OHP by ELISA (p <0.0001; r =0.95; serum 17OHP by ELISA = 1.70 × DBS 17OHP + 3.35); and the DBS 17OHP and serum 17OHP by LC-MS/MS (p <0.0001; r =0.95; serum 17OHP by LC-MS/MS = 1.31 × DBS 17OHP + 1.17).

### Morning PT and Morning 17OHP on Days 2 and 3

**Figure 1:**
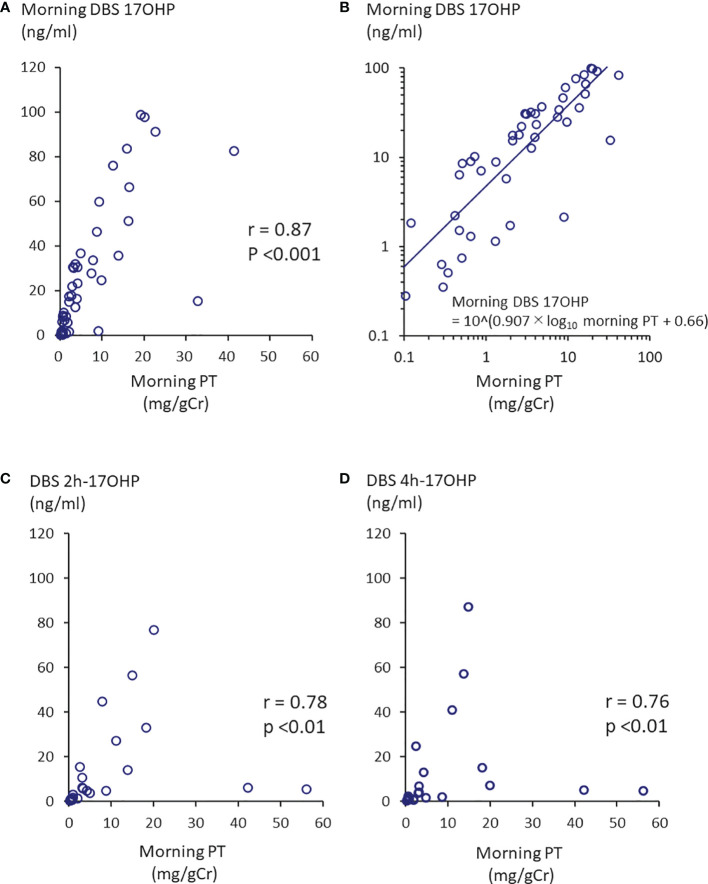
Correlation between morning PT and 17OHP. **(A)** Morning PT and morning DBS 17OHP showed a significant correlation (n =46, p <0.0001, r =0.87). **(B)** Regression formula between morning PT and morning DBS 17OHP. By log transformation, morning PT values were normally distributed. There were weaker correlations between **(C)** morning PT and 17OHP at 2 hours after morning GC administration (DBS 2h-17OHP) and **(D)** morning PT and 17OHP at 4 hours after morning GC administration (DBS 4h-17OHP).

The first morning urinary PT value (0h-PT) ranged from 0.10 to 41.3 mg/gCr (n=49; mean: 7.15 mg/gCr). Twenty-two of the 49 samples fell within the 10^th^ - 90^th^ percentile of the previous studies at 0.56-6.0 mg/gCr. The morning DBS 17OHP value (0h-17OHP) ranged from 0.28 to 99.0 ng/mL (n=48; mean: 28.4 ng/mL). There was a positive correlation between the 0h-PT and 0h-17OHP values (n =46; p <0.0001; r =0.87; **Figure 1A**). By log transformation, morning PT values were normally distributed but not 17OHP values were. The regression formula after log transformation was shown in **Figure 1B**. Extremely high PT values (37.2 and 41.3 mg/gCr) were obtained from one patient, whose 0h-17OHP values were 15.5 and 82.7 ng/mL for the respective time points. This patient’s condition was poorly controlled during the study period.

### Morning PT and 17OHP After Oral GC Administration (2h- and 4h-17OHP) on Day 1

The first morning urinary PT value (0h-PT) ranged from 0.12 to 56.1 mg/gCr (n=27; mean: 8.82 mg/gCr). The DBS 2h- and 4h-17OHP values ranged from 0.44 to 77.1 ng/mL (n=23; mean: 12.9 ng/mL) and from 0.30 to 87.2 ng/mL (n=23; mean: 12.8 ng/mL), respectively. There was a correlation between the 0h-PT and DBS 2h or 4h 17OHP values (**Figure 1C**; r =0.78, p <0.0001 and **Figure 1D**; r =0.76, p <0.0001).

### Optimal Range of 0h-17OHP Value

Based on the data of **Figure 1B**, morning DBS 17OHP, serum 17OHP by ELIZA, and LC-MS/MS value of 2.79-23.2 ng/mL, 7.94-42.8 ng/mL, and 4.71-31.6 ng/mL, respectively, was found to correspond to the 10^th^-90^th^ percentile of the first morning urine PT value (0.56-6.0 mg/gCr) demonstrated in our previous study (12) to be indicative of well-controlled disease. **Table 3** shows the range for each these 17OHP values corresponding to the 95% confidence interval of the mean first morning urine PT value (2.2-3.3 mg/gCr) in the well-controlled group.

**Table 3:** Optimal range of first morning PT and 0h-17OHP based on regression analysis.

	95% confidence interval	10^th^-90^th^ percentile
**Morning PT in previous study (mg/gCr)^#^**	2.2-3.3	0.56-6.0
**Calculated**		
** DBS 17OHP with ELISA (ng/mL)**	9.34-13.5	2.70-23.2
** Serum 17OHP with ELISA (ng/mL)**	19.2-26.3	7.94-42.8
** Serum 17OHP with LC-MS/MS (ng/mL)**	13.4-18.9	4.71-31.6

^#^Ref. (11, 12).

The optimal range equivalent to 4.0-12.0 ng/mL for morning serum 17OHP levels reported in previous studies (9, 10) was unable to be calculated because it was obtained using older, radioimmune assays, which were unavailable for use in the present study.

## Discussion

The present study is the first to compare morning urinary PT and blood 17OHP values. The first morning PT and 17OHP could be equivalent for biochemical monitoring because of the significant, positive correlation. On the other hand, it may be difficult to demonstrate the optimal 17OHP range after GC administration because weaker correlation between the 0h-PT and 2h- or 4h-17OHP values was found. **Table 3** shows the converted, optimal 0h-17OHP range, which is more reliable because the present study was designed specifically to assess the correlation between 0h-PT and 0h-17OHP and because the original, optimal morning urinary PT range was based on auxological data (11, 12).

A few studies have reported a correlation between blood 17OHP and urinary steroid metabolites (15, 16). Twenty-four-hour urinary steroid metabolites showing a significant correlation with 17OHP values were the PT/tetrahydrocortisone ratio, three 17OHP metabolites/three cortisol-cortisone metabolites ratio, 5α-pregnane-3α, 17α-diol-20-one (a backdoor pathway metabolite), among others. However, unlike our study, which used first morning urine samples, these previous studies used 24-hour urine samples. In most recently, Pussard et al. developed a novel LC-MS/MS method for urine measurement of 23-urinary steroids to show morning plasma and urinary 17-OHP were closely correlated (17).

Studies of the optimal range for biomarkers using auxological data, the gold standard index for 21-OHD, are limited in number (11, 12, 18–21). Most recently, Kamrath *et al.* suggested target values for urinary steroid metabolite excretions in children with 21-OHD based on their growth rate (21). They reported that the target range for androgen metabolite z-scores and hydrocortisone metabolite tetrahydrocortisol was 0.164 - 0.512 and <1480 µg/m^2^ body surface area/day, respectively. As described above, we previously demonstrated the optimal range for first morning urinary PT levels based on prepubertal auxological data (11, 12). Early morning urine collection is easier and more suitable for repetitive measurements than 24-hour urine collection. We have already shown a significant correlation in terms of PT between 24-hour urine and first morning urine samples (11). As far as cost is concerned, measuring PT alone than steroid metabolites in urine is more cost-effective.

The optimal range of 0h-17OHP in **Table 3** seems higher than the previously reported target range of 4-12 ng/mL (9, 10). The difference could be explained by differences in measurement assays and by the fact that the conventional optimal range were not based on auxological data.

In the clinical setting, measuring the first morning urinary PT value is more feasible and useful than measuring the morning blood 17OHP level for the obvious reason that urine collection is non-invasive and easier to perform than DBS. Robinson *et al.* (22) encountered difficulty in taking DBS samples from infants and young children. Further, performing an early morning blood collection before morning medication at a hospital is difficult; blood collection at the hospital outpatient clinic typically begins at 8:30AM whereas morning medication, in particular HDC, is ideally taken immediately after waking, given that 17OHP peaks from 4:00AM due to the circadian rhythm of ACTH (10). In fact, the blood collection time for 17OHP was not fixed at the outpatient department of most institutions (4) while no optimal 17OHP range was determined for the various times at which collection occurred. Finally, the first morning urine PT value can aid in monitoring disease control in a more integrated manner than the blood 17OHP value.

The present study also investigated the 17OHP level after each GC administration and revealed correlation with urinary PT. However, regression analysis was not investigated because following two reasons. First, the subjects included patients receiving two kinds of GC with differing pharmacokinetics, such as time to max. Second, even if only a single kind of GC, for example HDC, were administered, the peak plasma cortisol concentration after administration and the half-life of the GC would vary among individuals (23). In fact, the serum cortisol level at two hours after HDC administration in our subjects varied from 4.3 to 43.5 µg/dL (see **Table 1**).

A potential limitation of our study is that the DBS samples were collected by the patients themselves. As discussed above, DBS sampling at home may be difficult for non-medically-trained individuals, especially young pediatric patients, to perform (22). This difficulty may have affected the accuracy of our DBS 17OHP measurements. Therefore, the 17OHP in the DBS samples was measured twice using different spots, and the average value was used. In addition, the heterogeneity of the cohort may be another limitation. The present study contained various backgrounds including age, kind of GCs, and phenotype. If the purpose of study had been the utility of 17OHP and PT in monitoring of 21OHD, this would have been certainly a major problem. However, the aim of the present study was to explore the correlation between 17OHP and PT.

In conclusion, the first morning PT correlated more significantly with DBS 17OHP before morning medication. Measuring the first morning PT value may be more practical and useful to biochemically monitor 21-OHD.

## Data Availability Statement

The original contributions presented in the study are included in the article/**Supplementary Material**. Further inquiries can be directed to the corresponding author.

## Ethics Statement

The studies involving human participants were reviewed and approved by Institutional Ethics Committee of Tokyo Metropolitan Children’s Medical Center. Written informed consent to participate in this study was provided by the participants’ legal guardian/next of kin.

## Author Contributions

TI and YH designed the study. TI, MI, and TH collected the samples and data. TI analysed the data and wrote the first draft of the manuscript. YH reviewed/edited the manuscript and was responsible for all aspects of the research design and manuscript as the guarantor. All authors contributed to the article and approved the submitted version.

## Funding

This research was funded by JSPS KAKENHI, Grant Number 19K18009 awarded to TI.

## Conflict of Interest

The authors declare that the research was conducted in the absence of any commercial or financial relationships that could be construed as a potential conflict of interest.

## Publisher’s Note

All claims expressed in this article are solely those of the authors and do not necessarily represent those of their affiliated organizations, or those of the publisher, the editors and the reviewers. Any product that may be evaluated in this article, or claim that may be made by its manufacturer, is not guaranteed or endorsed by the publisher.
