# Supplementary material for: First Morning Pregnanetriol and 17-Hydroxyprogesterone Correlated Significantly in 21-Hydroxylase Deficiency
Source: Front Endocrinol (Lausanne). 2022 Jan 24;12:808254. doi: 10.3389/fendo.2021.808254 (PMC8820395; doi:10.3389/fendo.2021.808254)
Supplement: Supplementary file 1 [file Table_1.docx]

Supplementary Material

# Supplementary Tables

**Supplementary table 1. Clinical characteristics of the study cohort**

| No. | Sex | Age, year | Phenotype | Dosage of medication per body surface area, mg/m^2^ | | |
| --- | --- | --- | --- | --- | --- | --- |
|  |  |  |  | **HDC** | **DEX** | **FC** |
| 1 | F | 2 | NC | 5.1 | - | - |
| 2 | M | 3 | SW | 15.9 | - | 0.13 |
| 3 | F | 6 | SW | 22.7 | - | 0.09 |
| 4 | M | 7 | SW | 26.7 | - | 0.12 |
| 5 | M | 9 | SW | 16.6 | - | 0.02 |
| 6 | F | 9 | SW | 19.3 | - | 0.04 |
| 7 | F | 11 | SW | 12.9 | - | 0.04 |
| 8 | M | 11 | SW | 16.9 | - | 0.07 |
| 9 | F | 12 | SW | 23.3 | - | 0.04 |
| 10 | M | 13 | SW | 18.4 | - | 0.05 |
| 11 | M | 14 | SW | 18.9 | - | 0.03 |
| 12 | M | 17 | SW | 21.2 | - | 0.06 |
| 13 | M | 17 | SW | 27.0 | - | 0.07 |
| 14 | F | 20 | SW | 19.6 | - | 0.07 |
| 15 | M | 24 | SW | 19.5 | - | 0.03 |
| 16 | M | 26 | SW | 18.8 | - | 0.06 |
| 17 | F | 15 | SW | - | 0.24 | 0.06 |
| 18 | F | 20 | SW | - | 0.23 | 0.05 |
| 19 | F | 21 | SW | - | 0.41 | 0.06 |
| 20 | F | 23 | SW | - | 0.37 | 0.07 |
| 21 | F | 25 | SW | - | 0.3 | 0.07 |
| 23 | F | 27 | SW | - | 0.16 | - |
| 24 | M | 31 | SW | - | 0.34 | 0.03 |
| 25 | F | 33 | SW | - | 0.32 | 0.11 |

F, female; M, male; HDC, hydrocortisone; DEX, dexamethasone; FC, fludrocortisone
